# Maintaining Physical Activity Level Through Team-Based Walking With a Mobile Health Intervention: Cross-Sectional Observational Study

**DOI:** 10.2196/16159

**Published:** 2020-07-03

**Authors:** Yuri Hamamatsu, Hiroo Ide, Michiru Kakinuma, Yuji Furui

**Affiliations:** 1 Institute for Future Initiatives The University of Tokyo Tokyo Japan; 2 Healthcare and Wellness Division Mitsubishi Research Institute Inc Tokyo Japan

**Keywords:** mHealth, physical activity, walking, team-based, smartphone app

## Abstract

**Background:**

The health conditions of Japanese salespersons may be adversely affected by their lifestyle. Face-to-face or on-site health interventions are not convenient for salespersons because of their tendency for out-of-office sales. Previous studies showed that mobile health (mHealth) interventions (compared to usual practice) have great potential to promote physical activity. For Japanese salespersons, mHealth can offer additional convenience to change their physical activity habits because they can access the mHealth contents anytime and anywhere. However, the specific elements that are most important to maintain physical activity levels using an mHealth approach remain unclear.

**Objective:**

We aimed to identify elements that account for both a high average physical activity level and can help to prevent a decrease in physical activity during a 9-week intervention period.

**Methods:**

Salespersons were recruited from 11 Japanese companies. A team-based walking intervention was held from October to December 2018 (for a total of 9 weeks), during which the walking step data were recorded by smartphone apps. Average walking steps of each participant during the intervention and the difference in walking steps between the initial and the final week were respectively used as dependent variables. The effects of team characteristics (ie, frequency of communication with team members and team size) and behavioral characteristics (ie, number of days with recorded steps on the apps) on the average walking steps, and the difference in walking steps between the initial and the final week were estimated using multiple and multilevel regression analyses.

**Results:**

Of the 416 participants, walking step data of 203 participants who completed postintervention assessments were included in the analyses. Multiple regression analysis of the average walking steps showed that the number of days with recorded steps was positively correlated with the log-transformed average walking steps (β=.01, *P*<.001). Multilevel analysis of the average walking steps considering the company level estimated that the intraclass correlation coefficient was 37%. This means that belonging to the same company largely affected an individual’s average walking steps. Multiple regression analysis of the difference in walking steps showed that communication with team members once or twice a week correlated with preventing a decrease in walking steps from the initial to the final week (β=1539.4, *P*=.03), and being on a larger team correlated with a decrease in walking steps from the initial to the final week (β=–328.4, *P*=.01).

**Conclusions:**

This study showed that the elements accounting for high average walking steps and those preventing the decrease in walking steps from the initial to the final week differed. Behavioral characteristics correlated positively with average walking steps. Team characteristics (ie, regular communication and a smaller team size) significantly correlated with preventing a decrease in walking steps.

## Introduction

### Background

Previous studies have identified mobile Health (mHealth) as an effective and accessible tool that can be used by a large number of individuals, especially with the upsurge in mobile communication devices [1]. In addition, some studies have explored whether mHealth interventions would be more effective than usual interventions (eg, face-to-face, printed leaflets), or whether the change in target lifestyle from pre- to postintervention would be significant within the mHealth intervention group [2,3]. Previous reviews showed that mHealth has great potential to promote physical activity in a wide range of settings [4], including the workplace [5]. Maintaining good physical health or behavior change has been reported as hard to achieve [6,7], even if the study period is relatively short (eg, 1-3 months) [8]. In particular, some mHealth intervention studies consistently showed a decline in physical activity levels over time [9,10]. However, no study has identified the significant elements required to maintain the physical activity level in an mHealth intervention.

### Health Condition Among Japanese Salespersons

Being a salesperson is one of the representative job types in Japan. According to the 2015 population census [11], salespersons (7,410,702) accounted for 14% of all employed Japanese individuals, and ranked as the fourth largest among 12 job categories. Therefore, an intervention program focusing on salespersons can have an important role in health promotion at Japanese worksites.

The number of companies that are strategically promoting “Health and Productivity Management,” an approach that considers the health management of employees from a corporate management perspective, has been increasing in Japan [12]. The physical activity level of the Japanese population aged 20 to 59 years (working population) in 2018 was lower than that of the average Japanese population; the percentage of Japanese men and women reporting a regular exercise habit was 31.8% and 25.5%, respectively, and was 5%-10% lower among people aged 20 to 50 years [13]. Particularly at Japanese worksites, the health conditions of salespersons may be more adversely affected than those of others due to their lifestyle. For instance, salespersons are more likely to work long hours, become inactive, and skip meals [14], partly because their customer’s schedule comes first. It is not convenient for salespersons to use a face-to-face or an on-site health intervention such as providing healthy company cafeteria meals, because of their tendency toward out-of-office sales. Therefore, mHealth can offer additional convenience to change the physical activity habits of Japanese salespersons because they can access the mHealth contents anytime and anywhere. However, to the best of our knowledge, there has been no mHealth research that has focused specifically on Japanese salespersons.

### Team-Based and Competitive Intervention Program

Previous studies have suggested that a team-based competitive approach would be effective to improve individual health-related behaviors and health outcomes. Shape Up Rhode Island, a statewide team-based campaign to promote weight loss and walking, showed that changes in weight were similar among teammates, and being on a more active team was associated with a greater increase in activity for individual members [15,16]. Accordingly, we expect that being a part of a team can improve the individual physical activity of team members through supportive interactions that encourage healthy behaviors [17,18]. In addition, it can be anticipated that people in a competitive environment use rankings as motivation to work toward their individual goals [18]. Therefore, we expect that an intervention program based on a team-based competitive approach would also be effective in targeting salespersons.

In this study, we conducted a team-based competitive walking intervention for Japanese salespersons via a smartphone app. The participants were required to record their daily step counts via the smartphone app. The participants could also check their individual daily step counts and their team’s weekly ranking via the smartphone app.

### Goal of this Study

This observational study aimed to identify elements that account for maintenance of physical activity during the intervention. Two different approaches that could be used to estimate the extent to which participants maintain their physical activity levels are calculating the average physical activity level and the amount of change of physical activity from the initial to the final period during the intervention. Therefore, the purpose of our study was two-fold, namely: (1) to estimate the effect of team and individual behavioral characteristics on the average walking steps of each participant, and (2) to examine the effect of team and individual behavioral characteristics on the difference in walking steps between the initial and final week of the program.

## Methods

### Contents of the “Walking Intervention”

We held the “Walking Intervention” to promote walking activity via smartphone apps at 11 companies representing the financial, manufacturing, and pharmaceutical industries.

To recruit participants, we distributed the original leaflets to sales departments at each company. We did not provide any participant incentives for applicants at the recruitment stage of this study. Applicants who were regular employees, working in the sales department of each company, and who could carry smartphones with them during working hours to record their walking steps were eligible to participate in this intervention. The decision to include applicants under medical treatment in this intervention was officially determined at the discretion of a physician. We registered 416 participants from September to October 2018. A total of 85 teams of 3 to 8 members each were formed. The team was formed by self-selected members or by an intervention manager at each company who recruited participants from their own worksites.

Before the Walking Intervention started, we explained to each participant the health benefits of walking, but the participants did not have to set individual daily walking goals (eg, 8000 steps a day). Since the participants’ worksites and residences geographically varied, we did not have any joint event to promote the participants’ walking behavior. In addition, we did not officially provide any messages to the participants recommending them to walk more.

Since salespersons are likely to be out of the office for sales and need an easy-to-participate program that can be conducted anytime and anywhere, we held the intervention via smartphone apps. The participants downloaded the app CARADA (MTI Ltd, Tokyo, Japan) to allow them to check their daily walking steps. Individual and team rankings were calculated weekly by the walking step counts displayed in the app.

**Figure 1:**
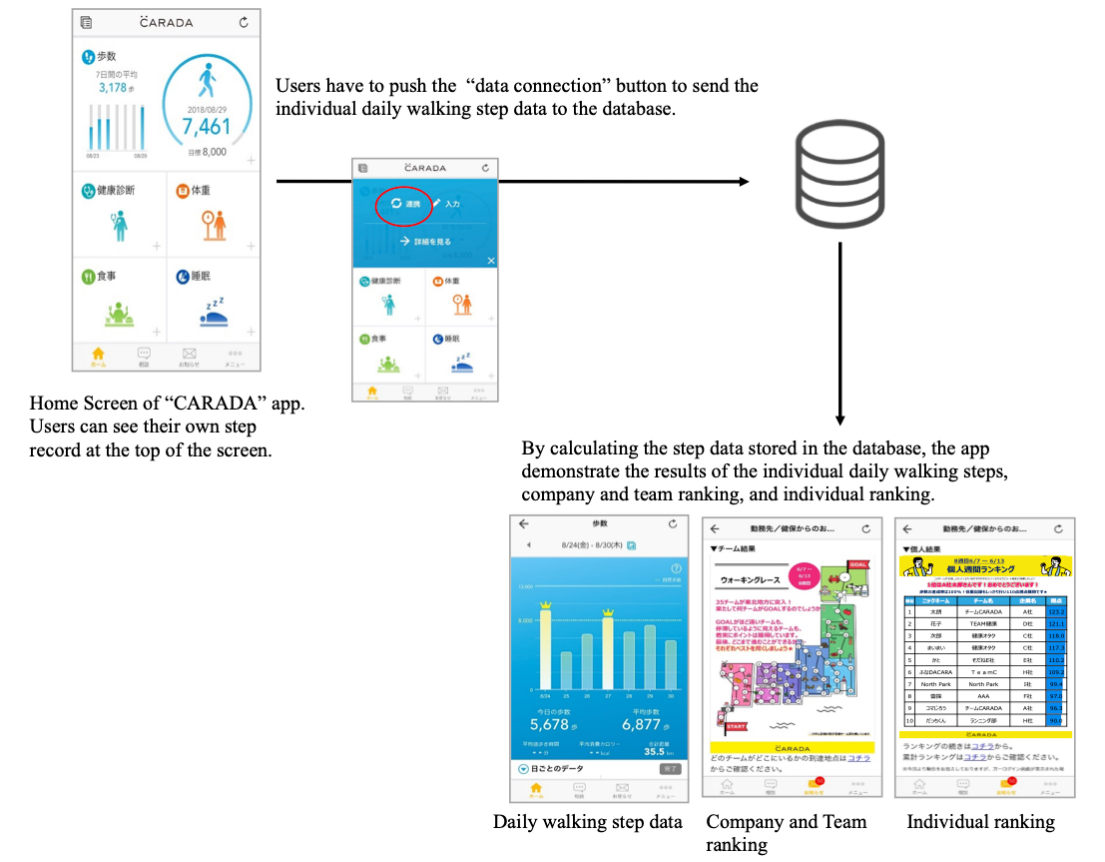
Features of the CARADA app.

Figure 1 demonstrates the features of the CARADA app. The app can automatically record the participants’ daily walking steps and shows their step records at the top of the home screen. To send their daily walking step data to the database, the participants have to push the “data connection” button manually. The app displays the results of the individual daily walking steps, along with the company, team, and individual rankings by calculating the step data stored in the database. Based on previous studies indicating the effectiveness of social comparison [18], we expected that the participants would be motivated by checking the company, team, and individual ranking results.

We held a seminar at each company before starting the walking intervention during which the intervention manager explained the purpose of the intervention, the rules, and how to download the CARADA app. Seminars were held at a total of 7 companies; the other 4 companies did not have the chance to gather the participants together before the intervention. Therefore, for the companies that did not have a seminar in advance, we provided materials that included the same information as provided in the seminar: the purpose of the intervention, the rules, and how to download the CARADA app.

The intervention was held from October 17 to December 17, 2018 (total of 9 weeks or 62 days). One company had a 2-week delay from October 31 to December 31, 2018.

### Postintervention Assessment

We administered questionnaires to the participants after the intervention (January 2019) via the CARADA app to assess the frequency of communication between team members, daily means of transportation during working hours, the reason for participating in this intervention, and the applicability of an app-based walking intervention. According to previous studies [15-18], we hypothesized that some factors (eg, good team relationship) would be essential for the success of a worksite health intervention. Therefore, we developed the questionnaires based on the hypothesis and through discussion with the intervention managers (see Multimedia Appendix 1). To reduce the burden on the participants, we did not insist that they answer the questionnaires both prior to and after the intervention.

### Statistical Analyses

Because the distribution of average walking steps was right-skewed, we could not apply a parametric analysis that requires a normal distribution. Therefore, the Wilcoxon rank-sum test or Kruskal-Wallis rank-sum test was used to compare the median of average walking steps between the explanatory categorical variables. One-way analysis of variance (ANOVA) was used to compare the average difference in walking steps between the initial and final week between the explanatory categorical variables.

We performed analyses to estimate the effects of team and behavior characteristics on average walking steps and the difference of walking steps between the initial and the final week. The following team characteristics were included: team size (number of participants in a team, ranging from 3 to 8 members) and the frequency of communication with team members (rarely=reference, once or twice a month, once or twice a week, three or four times a week, every day). As a behavioral characteristic, the number of days with recorded steps of each participant was examined.

We first performed multiple regression and multilevel analyses to examine the effect of team and individual behavioral characteristics on the average walking steps, and then performed an additional multiple regression analysis to examine the effect of team and individual behavioral characteristics on the difference in walking activity between the initial and the final week throughout the program.

For the first analyses, the average walking steps of each participant during the intervention was the dependent variable. Multiple regression analysis was performed to examine the relationship of team and behavioral characteristics with average walking steps. As the degree of health consciousness and motivation for physical activity were considered to likely differ among the 11 companies, we subsequently applied the random intercept model to consider the random effect of the company to which each participant belonged. In addition, we used the business category (finance, manufacturing, and pharmaceutical) as a confounding variable because the lifestyle and work style among salespersons could vary according to business category. The average walking steps of each participant was log-transformed as a dependent variable, because the values were always above zero and the distribution was right-skewed.

For the second analysis, the difference in walking steps between the initial (ie, first and second week) and the final week (ie, ninth week) for each participant was the dependent variable. The difference in walking steps between the initial and the final week was calculated by subtracting the average walking steps for the final week from that of the initial week. Multilevel analysis was also used because the data had a clustered structure (ie, step count data of participants within companies). However, because the eligible data for the analysis of difference in walking steps included that of a company with step counts from only one participant, we did not apply the multilevel analysis for the difference in walking steps.

We used the following factors as confounding variables: sex (male=1, female=reference), age (years), daily means of transportation during working hours (1=use of a public transportation system, not using public transportation=reference) or walking (walking=1, not walking=reference), the reason for deciding to participate in the intervention (voluntary participation=1, invitation from their colleagues or boss=reference), the business category to which the participants belonged (finance=reference, manufacturing, pharmaceutical), and whether the company to which the participants belonged had a seminar before the intervention (having a seminar=1, not having a seminar=reference). We examined the correlation coefficients of all explanatory variables. All correlation coefficients were between –0.5 and 0.5, and there were no strong correlations observed among the explanatory variables.

Statistical analyses were performed using R 3.5.1 software with the dplyr [19], ggplot2 [20], and lme4 [21] packages. A *P* value <.05 was considered statistically significant.

### Ethical Approval

This study was conducted with payouts from the Ministry of Health, Labor, and Welfare, Japan. The University of Tokyo Human Research Ethics Committee approved this study (18-213). All participants received a written and/or verbal explanation of the intervention requirements before providing consent. We provided an informed consent form when the participants downloaded the CARADA app.

## Results

### Recruitment and Participants

**Figure 2:**
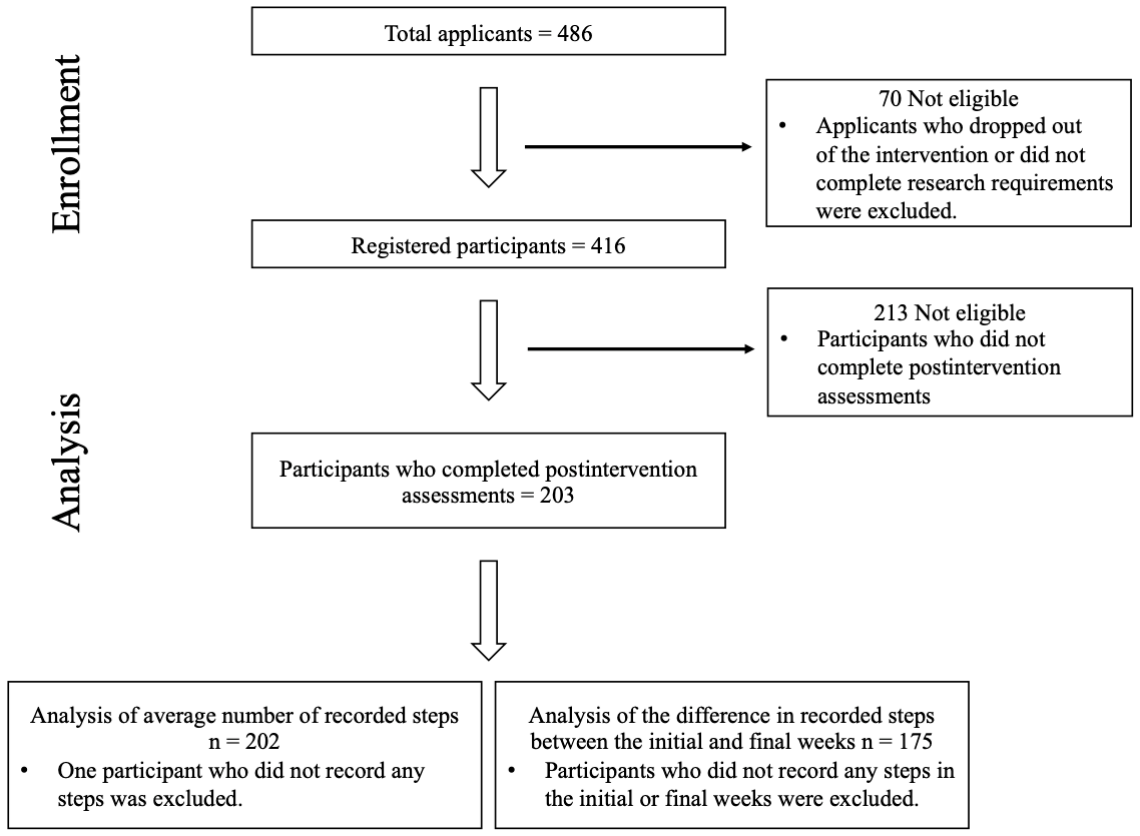
Study flow diagram.

Of the 486 people who applied to participate in the walking intervention, 70 were not eligible for official participation because they either dropped out of the intervention or did not comply with the research requirements. Therefore, we officially registered 416 participants for the walking intervention. We included the walking step data of 203 participants who completed the assessments after the intervention in the final analyses (Figure 2). We did not include any participants with incomplete data in the analysis (n=213) who did not complete the final assessment, because some essential explanatory variables were obtained only from the final assessment. For example, it was not possible to ascertain the exact subjective team evaluation (eg, frequency of communication with team members) from the noncompleters. Therefore, if a team was composed of only noncompleters, we could not analyze that team in this study.

### Characteristics of Participants

Table 1 summarizes the characteristics of the final 202 participants included in the analyses on the average walking steps during the intervention. We excluded one of the 203 participants who completed the assessments after the intervention from this analysis because they failed to record any steps during the intervention. Table 2 shows the characteristics of 175 participants included in the analyses with respect to the difference in walking steps between the initial and the final week (28 of 203 participants who did not record any steps at the initial or during the final week were excluded).

The sample predominantly comprised men for both the analysis on the average walking steps and the analysis on the difference of walking steps. The majority of participants communicated with their team members once or twice a week among the five communication categories. The average team size was 5.8. The number of recorded step days was 54.7 for the analysis on the average walking steps and was 58.5 days for the analysis on the difference of walking steps (Table 1 and Table 2).

**Table 1:** Characteristics of participants for the analysis on average walking steps during the intervention (N=202^a^).

Characteristic		Value
Sex (male), n (%)		159 (78.7)
Age (years), mean(SD)		41.3 (8.91)
**Daily means of transportation, n (%)**		
	Public transportation system=no	131 (64.9)
	Public transportation system=yes	71 (35.1)
	Walking=no	137 (67.8)
	Walking=yes	65 (32.2)
Voluntary participation, n (%)		141 (70.1)
**Business category, n (%)**		
	Finance	90 (44.6)
	Manufacturing	22 (10.9)
	Pharmaceutical	90 (44.6)
Having a seminar before the intervention, n (%)		154 (76.2)
**Frequency of communication with team members, n (%)**		
	Rarely	31 (15.4)
	Once or twice a month	36 (17.9)
	Once or twice a week	73 (36.3)
	Three or four times a week	28 (13.9)
	Every day	33 (16.4)
	Did not answer	1 (0.5)
Team size, mean (SD)		5.8 (1.62)
Number of days with recorded steps, mean (SD)		54.7 (12.44)

^a^One participant who did not record any steps at all was excluded from the total 203 participants.

**Table 2:** Characteristics of participants for the analysis on the difference of walking steps between the initial and the final week (N=175^a^).

Characteristic		Value	
Sex (male), n (%)			136 (77.7)
Age (years), mean (SD)			41.9 (9.04)
**Daily means of transportation, n (%)**			
	Public transportation system=no		108 (61.7)
	Public transportation system=yes		67 (38.3)
	Walking=no		115 (65.7)
	Walking=yes		60 (34.3)
Voluntary participation, n (%)			122 (70.1)
**Business category, n (%)**			
	Finance		78 (44.6)
	Manufacturing		17 ( 9.7)
	Pharmaceutical		80 (45.7)
Having a seminar before the intervention (%)			132 (75.4)
**Frequency of communication with team members (%)**			
	Rarely		22 (12.6)
	Once or twice a month		30 (17.2)
	Once or twice a week		66 (37.9)
	Three or four times a week		26 (14.9)
	Every day		30 (17.2)
	Did not answer		1 (0.6)
Team size, mean (SD)			5.8 (1.60)
Number of days with recorded steps, mean (SD)			58.5 (6.49)

^a^Twenty-eight participants who did not record any steps in the initial or final week were excluded from the total 203 participants.

Multimedia Appendix 2 shows the results of the comparison between the completers (n=203), who completed the final assessment, and the noncompleters (n=213), who did not complete the final assessment, in terms of age, sex, and the average recorded walking steps. The completers were significantly older, walked more, and the proportion of female completers was higher than that of noncompleters.

### Descriptive Statistics

**Figure 3:**
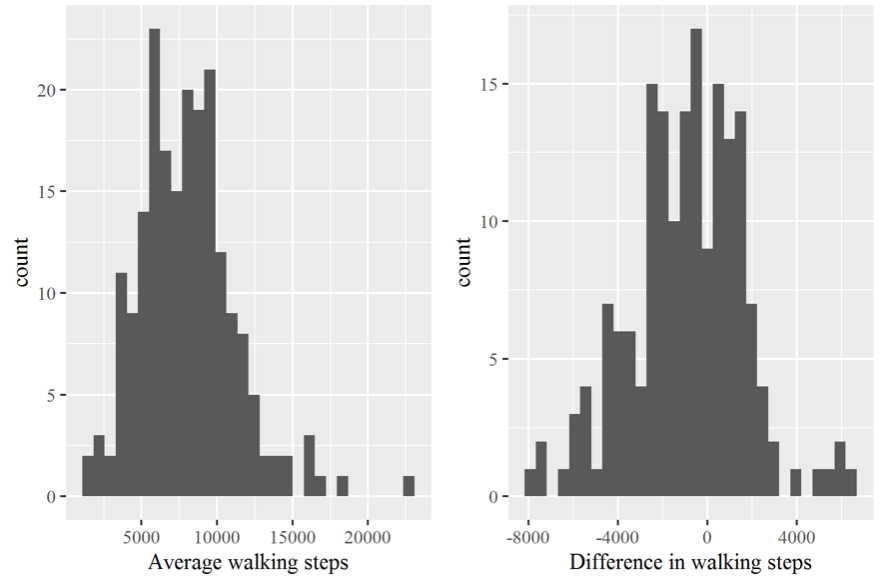
Distribution of the average walking steps and difference in walking steps between the initial and the final week for each participant.

Figure 3 shows the distributions of the average walking steps and difference in walking steps between the initial and the final week. The distribution of the average walking steps was right-skewed, and the distribution for the difference in walking steps tended to be normal.

The mean and median (IQR) average walking steps was 8024 and 7908 (5710-9819), respectively, with a range of 1724 to 22,965. The Shapiro-Wilk normality test showed a significant effect (*W*=0.96, *P*<.001). The mean and median (IQR) difference in walking steps between the initial and the final week was –898.0 and –760.4 (–2462.6 to 878.8), respectively, with a range of –7908.5 to 6429.2. The Shapiro-Wilk normality test was not significant (*W*=0.99, *P*=.19).

Table 3 shows the Wilcoxon or Kruskal-Wallis rank-sum test results for the average walking steps of each participant for each categorical variable. The average walking steps differed significantly between the following variables: using the public transportation system or walking, the company’s business category to which the participants belonged, and having a seminar before the intervention. The average walking steps and age were significantly correlated (Pearson correlation coefficient 0.23, *P*=.001). The average walking steps was also significantly correlated with the number of days with recorded steps (Pearson correlation coefficient 0.37, *P*=.001). Team size did not significantly correlate with average walking steps. The participants who did not have a seminar recorded significantly higher average walking steps than those who did receive the seminar in advance (Table 3).

**Table 3:** Average walking steps for each explanatory variable.

Categorical variables		n	Median (IQR) average steps^a^	*P* value^b^
**Sex**				.08
	Female	43	8404 (6888-10,228)	
	Male	159	7623 (5610-9701)	
**Use of public transportation system**				<.001
	No	131	6775 (5350-8618)	
	Yes	71	9527 (8170-11,185)	
**Walking**				<.001
	No	137	7025 (5576-9183)	
	Yes	65	9149 (7570-11,003)	
**Voluntary participation**				.86
	No	60	7806 (5572-9799)	
	Yes	141	7910 (5782-9831)	
	Did not answer	1	9333 (N/A^c^)	
**Business category**				.003
	Finance	90	8363 (5961-10,370)	
	Manufacturing	22	9235 (7864-9936)	
	Pharmaceutical	90	6778 (5391-9123)	
**Taking a seminar before the intervention**				<.001
	No	48	8816 (8040-10,346)	
	Yes	154	6967 (5391-9468)	
**Frequency of communication with team members**				.27
	Rarely	31	8058 (5667-9626)	
	Once or twice a month	36	7154 (4505-9251)	
	Once or twice a week	73	7750 (5722-10,158)	
	Three or four times a week	28	7385 (5319-9151)	
	Every day	33	8529 (6602-10,333)	
	Did not answer	1	9333 (N/A)	

^a^The distribution of average walking steps of each participant was right-skewed; therefore, the medians of average walking steps with IQR are shown for each categorical variable.

^b^The *P* values for categorical variables were estimated by the Wilcoxon or Kruskal-Wallis rank-sum test.

^c^N/A: not applicable.

One-way ANOVA results for the difference in walking steps between the initial and the final week for each categorical variable demonstrated that participants who used public transportation systems or walked for their daily transportation recorded a significantly smaller difference in walking steps between the initial and the final week than those who did not use public transportation systems or did not walk for daily transportation. There were no significant correlations between any of the continuous variables and the difference in walking steps (Table 4).

**Table 4:** Difference in walking steps between the initial and the final week for each explanatory variable.

Categorical variables		n	Mean (SD) of difference	*P* value^a^
**Sex**				.22
	Female	39	–453 (2638)	
	Male	136	–1026 (2542)	
**Use of public transportation system**				.006
	No	108	–1315 (2671)	
	Yes	67	–226 (2252)	
**Walking**				.01
	No	115	–1241 (2519)	
	Yes	60	–240 (2550)	
**Voluntary participation**				.72
	No	52	–1017 (2284)	
	Yes	122	–865 (2690)	
	Did not answer	1	1216 (N/A^b^)	
**Business category**				.79
	Finance	78	–762 (2325)	
	Manufacturing	17	–846 (1786)	
	Pharmaceutical	80	–1042 (2926)	
**Having a seminar before the intervention**				.10
	No	43	–333 (2186)	
	Yes	132	–1082 (2661)	
**Frequency of communication with team members**				.42
	Rarely	22	–1797 (2220)	
	Once or twice a month	30	–1082 (3244)	
	Once or twice a week	66	–633 (2446)	
	Three or four times a week	26	–995 (1751)	
	Every day	30	–624 (2891)	
	Did not answer	1	1216 (N/A)	

^a^The *P* values for categorical variables were calculated by one-way analysis of variance.

^b^N/A: not applicable.

### Multiple Regression and Multilevel Analyses

Table 5 shows the multiple regression analysis results for the average walking steps (multiple *R^2^*=0.36; adjusted *R^2^*=0.31). The behavioral characteristic (number of days with recorded steps) was positively correlated with the log-transformed average walking steps (β=.01, *P*<.001).

**Table 5:** Multiple regression analysis for factors influencing average walking steps during the intervention^a^ (N=192^b^).

Variables		β^c^	SE	*P* value
Intercept		8.05	0.22	<.001
Sex (male=1)		–.02	0.07	.80
Age (years)		.01	0.003	.02
Daily means of transportation (using public transportation system=1)		.18	0.07	<.001
Daily means of transportation (by walking=1)		.04	0.06	.50
Voluntary participation (yes=1)		.04	0.07	.54
**Business category**				
	Finance (reference)	N/A^d^	N/A	N/A
	Manufacturing	.09	0.1	.36
	Pharmaceutical	–.09	0.08	.27
Having a seminar before the intervention (yes = 1)		–.07	0.08	.41
**Frequency of communication with team members**				
	Rarely (reference)	N/A	N/A	N/A
	Once or twice a month	–.08	0.09	.38
	Once or twice a week	–.02	0.08	.80
	Three or four times a week	–.03	0.1	.77
	Every day	.03	0.1	.75
Team size		–.01	0.02	.48
Number of days with recorded steps		.01	0.002	<.001

^a^The participants’ average steps were log-transformed.

^b^Participants with missing data for explanatory variables were excluded from the analysis.

^c^Partial regression coefficients are reported.

^d^N/A: not applicable.

Table 6 shows the multilevel analysis results for the average walking steps according to the participants’ company level. The intraclass correlation coefficient (ICC) was 37% in the null model (0.07 [variance of company]/0.12 [variance of residual]+0.07 [variance of the company]), indicating that belonging to the same company largely affected an individual’s average walking steps. The Akaike Information Criterion value for the null model was 178.93 and was 207.3 for Model 1. In this multilevel analysis, the number of days with recorded steps still showed a significant positive correlation with the log-transformed average walking steps.

**Table 6:** Multilevel analysis results for average walking steps during the intervention^a^ (N=192^b^).

Variables		β^c^	SE	*P* value
(Intercept)^d^		8.21	0.28	<.001
Sex (male=1)		.12	0.07	.07
Age (years)		.002	0.003	.43
Daily means of transportation (using public transportation system=1)		.14	0.07	.04
Daily means of transportation (walking=1)		–.01	0.06	.82
Voluntary participation (yes=1)		.06	0.07	.38
**Business category**				
	Finance (reference)	N/A^e^	N/A	N/A
	Manufacturing	.05	0.20	.82
	Pharmaceutical	–.16	0.21	.48
Having a seminar before the intervention (yes=1)		–.10	0.20	.63
**Frequency of communication with team members**				
	Rarely (reference)	N/A	N/A	N/A
	Once or twice a month	–.05	0.08	.56
	Once or twice a week	–.01	0.08	.90
	Three or four times a week	–.08	0.09	.38
	Every day	.06	0.09	.52
Team size		–.0008	0.02	.96
Number of days with recorded steps		.01	0.002	<.001

^a^The participants’ average steps during the intervention were log-transformed.

^b^Participants with missing explanatory variable data were excluded from the analysis.

^c^Partial regression coefficients are reported.

^d^Intercept of null model=9.02 (–0.09)

^e^N/A: not applicable.

Table 7 shows the multiple regression analysis results for the difference in walking steps between the initial and the final week (multiple *R^2^*=0.13, adjusted *R^2^*=0.04). For the team characteristics, significant correlations were observed between having communication with team members once or twice a week and preventing a decrease in walking steps from the initial to the final week. Being on a larger team was also correlated with a decrease in walking steps from the initial to the final week.

**Table 7:** Multiple regression model results for the difference in walking steps between the initial and the final week (N=167^a^).

Variables		β^b^	SE	*P* value
(Intercept)		2810.2	2331.2	.23
Sex (male=1)		–659.5	526.9	.21
Age (years)		–11.2	23.8	.64
Daily means of transportation (using public transportation system=1)		1051.7	515.1	.04
Daily means of transportation (walking=1)		613.1	487.9	.21
Voluntary participation (yes=1)		–316.8	558.7	.57
**Business category**				
	Finance (reference)	N/A^c^	N/A	N/A
	Manufacturing	–11.9	767.7	.99
	Pharmaceutical	688.2	599	.25
Having a seminar before the intervention (yes=1)		–453.6	597.5	.45
**Frequency of communication with team members**				
	Rarely (reference)	N/A	N/A	N/A
	Once or twice a month	1041.4	741.9	.16
	Once or twice a week	1539.4	683.4	.03
	Three or four times a week	1142.6	775.4	.14
	Every day	1143.8	777.7	.14
Team size		–328.4	130.8	.01
Number of days with recorded steps		–39.4	32.3	.23

^a^The participants with missing explanatory variable data were excluded from the analysis.

^b^Partial regression coefficients are reported.

^c^N/A: not applicable.

## Discussion

### Principal Results

The average walking steps had a significant positive correlation with the individual behavioral characteristic (number of days with recorded steps). However, preventing a decrease in walking steps from the initial to the final week had significant correlations with two team characteristics (having communication with team members once or twice a week and being on a smaller sized team).

Previous studies have suggested that a positive change in physical activity was most likely to occur at the beginning of the program, and the physical activity level tended to decrease from the beginning to later periods [9,10]. The present study showed that the elements contributing to recording a higher number of average walking steps and those contributing to preventing a decrease in walking steps from the initial to the final week were different. The intervention for individual behavior (eg, offering an easy activity tracking tool) was effective in enhancing the basic physical activity level. The intervention for team relationships (eg, providing support for team members via some communication tools) was effective in preventing a decrease in physical activity level during the 9-week intervention period.

### Average Walking Steps

The number of days with recorded steps was positively correlated with the average walking steps throughout the intervention. Recording step counts via smartphone apps can offer an easy way of monitoring the steps, and thus provide direct feedback on daily walking patterns. Previous studies have reported that use of a self-monitoring technique was associated with a positive change in health-related behavior and health outcomes [22,23]. For example, self-weighing led to success with weight loss; furthermore, with a greater number of days that participants monitored their own weight, there was a greater decrease in weight [23]. Intervention programs using self-monitoring methods also showed great potential to increase physical activity [22]. Feedback tools can be effective for increasing physical activity; Kang et al [24] showed that using a pedometer with a feedback tool significantly increased the participants’ motivation to become more active.

Smartphone apps can provide more effective self-monitoring and feedback tools than traditional pedometers [25]. For example, smartphone apps can be equipped with visible feedback content, which is not possible with traditional pedometers. It may be more convenient to record and monitor daily step counts via smartphone apps than traditional pedometers, as people usually carry smartphones with them throughout the day. In addition, smartphone apps can offer more accessible self-monitoring tools than a website-based program [26]. Therefore, it is speculated that the number of days with recorded steps in this study might have been amplified by using smartphone apps with strong self-monitoring and feedback features.

In the multilevel analysis on the average walking steps, the ICC was 37% and being in the same company largely explained the individual variance in average walking steps. It is possible that a company that employed more healthy salespersons happened to have a healthy culture and more motivation toward physical activity as a whole. However, considering the results of previous studies showing that community culture has a significant impact on individual health-related behaviors and health outcomes [27-29], the causal relationship between health outcomes of individual employees and the degree of health consciousness at the company level could be in the opposite direction. This study showed that participants who did not have a seminar before the intervention recorded a higher average number of walking steps. One possible reason is that companies that consider their employees to not have a healthy lifestyle and promote Healthy and Productivity Management [12] might have encouraged the participants to take a seminar to further motivate them. However, the seminar was not effective in improving the employees’ behavior. We can assume that interventions to raise awareness about health that are administered company-wide can result in greater success in the change in individual employees’ health outcomes.

### Difference in Walking Steps

In the analyses on the difference of walking steps between the initial and the final week, a smaller team size correlated with preventing a decrease in walking steps throughout the intervention. It has been suggested that a social network and social support can contribute to increased physical activity levels [30,31]. We assumed that the participants in smaller teams would more easily be able to communicate with each other, and that the positive social support effect was more likely to occur in smaller rather than in larger teams.

Having communication with team members once or twice a week also correlated with preventing a decrease in walking steps from the initial to the final week. Previous studies showed that having a social interaction could reduce participants’ attrition [32]. It is possible that having regular communication built social support among team members, which contributed to preventing a decrease in walking steps from the initial to the final week. However, there was no dose-response relationship between the frequency of communication and prevention of a decrease in walking steps. Having communication more than once or twice a week did not correlate with preventing a decrease in walking steps. One possible explanation is that the effect of social support on physical activity performance was influenced by personality type [33]. Some participants may feel encouraged to do more walking, whereas others may feel pressured from social support. Therefore, this finding may indicate that a moderate level of communication is needed to promote physical activity.

### Limitations

One of the potential limitations of this study is that we included participants who voluntarily decided to participate in this walking intervention; thus, the sample population might have been comparatively more health-conscious than the general population. We excluded 213 participants from the analyses who did not complete the assessment. In addition, we conducted the assessment via a smartphone app; therefore, it is possible that participants who were unfamiliar with the use of smartphone apps could not properly respond to the assessment. There is a need for further examination on whether the effectiveness of self-monitoring and feedback via smartphone apps that was observed in this study can also be applied to people who are unfamiliar with the use of smartphone apps. The assessment was conducted after the intervention, raising the possibility that only the people who were motivated by the walking intervention might have answered the assessment questionnaire. The people who were highly motivated may have been the participants who were more satisfied with this intervention because of their team relationship or their success with walking behavior. In fact, the average number of recorded walking steps among the completers was higher than that of noncompleters. It is possible that only the people with a high number of recorded walking steps or that had good communication with team members might have been included in this analysis. The same bias could have occurred among the 28 of the 203 participants who did not record any steps in the initial week or during the final week, and were therefore excluded in the analyses due to a lack of difference in walking steps between the initial and the final week.

Additionally, the sample was predominantly comprised of men. According to the population census of 2015 [11], the proportion of men among salespersons was about 56%. The proportion of men in this study was larger than that indicated from the census result. One of the possible reasons for this discrepancy is that the industries selected to focus on in this study tend to employ more male salespersons than female salespersons; for example, the proportion of male salespersons in the manufacturing industry (including the pharmaceutical industry) was over 80% and that in the financial industry was about 50% [11]. Therefore, a wide range of industries should be targeted to generalize these results in future studies.

The number of days with recorded steps might also reflect the participants’ motivation for walking; that is, participants with a more inherent motivation to walk would record a higher number of days with steps. This could have resulted in reduced effectiveness of self-monitoring and feedback using smartphone apps compared to expectation.

Another limitation is that the study period was shorter than that used in other studies focusing on the long-term intervention effect on physical activity. For example, Buckingham et al [5] reviewed the studies regarding an mHealth intervention for physical activity and reported that a significant mHealth effect on physical activity was largely observed from 1 month to 12 months after the beginning of the intervention. Although our study period was only 9 weeks, we expected that not all of the participants would maintain their physical activity level during this period. For example, Boyce et al [8] reported that the greatest change in mean BMI occurred within the first few weeks (ie, 4 weeks) and then plateaued until the end of intervention (ie, at week 12). Therefore, we expected that the physical activity level would decline in most participants, while some would maintain the increased physical activity level. Our aim was to investigate the specific factors that would most contribute to these differences in physical activity levels. Therefore, follow-up research after the intervention (eg, 12 months) should be conducted to assess the long-term effects similar to previous studies.

This study used a multilevel analysis to consider the company effect of this clustered data structure. We also tried to consider the clustered effect of a team, but as some teams had only one eligible participant for analysis, we could not apply the multilevel analysis considering the team effect. We checked the correlation between all of the explanatory variables (including team variables). All correlation coefficients were between –0.5 and 0.5, and the observed explanatory variables were numerically independent. However, it is possible that the clustered effect of a team was not sufficiently investigated.

As this study used a cross-sectional design, we cannot infer causal relationships between the individual recorded steps and team or behavioral characteristics. We did our best to adjust for possible confounders using statistical analysis, but since this study was not a randomized controlled trial, there are many unmeasured confounders that could also explain the results. Therefore, we cannot simply generalize the results from this study.

### Conclusion

This study showed that the elements that accounted for raising the average number of walking steps and those that accounted for preventing a decrease in walking steps from the initial to the final week were different. The behavioral characteristic (number of days with recorded steps via smartphone apps) was positively correlated with the average number of walking steps. Team characteristics (having regular communication once or twice a week and a smaller team size) were significantly correlated with preventing a decrease in walking steps. Considering the above factors, we can develop more effective mHealth programs focusing on salespersons, which can be used for maintenance of high physical activity levels.

## Appendix

Multimedia Appendix 1 Questionnaires for assessing motivations and experience after the walking intervention.

Multimedia Appendix 2 Comparison of characteristics between completers and noncompleters in terms of age, sex, and average recorded walking steps.

## Abbreviations

ANOVA: analysis of variance
ICC: intraclass coefficient
mHealth: mobile health
