# Supplementary material for: Maintaining Physical Activity Level Through Team-Based Walking With a Mobile Health Intervention: Cross-Sectional Observational Study
Source: JMIR Mhealth Uhealth. 2020 Jul 3;8(7):e16159. doi: 10.2196/16159 (PMC7367537; doi:10.2196/16159)
Supplement: Multimedia Appendix 1 [file mhealth_v8i7e16159_app1.docx]

Questionnaires for Assessing Motivations and Experience After the Walking Intervention

Q1 This section asks you questions about your company or work.

Please select from the options that best describes your work

1. On average, how many hours do you work a day (including overtime)?

Less than 4 hours

4 to 6 hours

6 to 8 hours

8 to 10 hours

10 to 12 hours

12 to 14 hours

14 hours or more

1. How much of your time do you spend sitting down during work? (Examples: at your desk, in your car seat)

　　　　　　　　　　　　　％

1. How much experience do you have in sales? (Please answer based on the total years of experience in sales including those with previous employers).

Less than 1 year

1 to 2 years

2 to 5 years

5 to 10 years

10 to 20 years

20 to 30 years

30 years or more

1. What means of transportation do you often use often in your daily work? (Multiple options can be selected)

Bicycle

Motorcycle

Public transportation (Trains and buses)

Walk

Daily work rarely requires travel

Other (Please describe specifically)

1. Please rate your work performance in the last four weeks, assuming that 100% indicates your maximum performance when you are not ill, injured or feeling unwell.

　　　　　　　　　　　　％

1. Please select an option to rate how well the following statements describe your work conditions and performance. (5 items)

6－1 Our workplace has good team spirit.

Agree　　　　Somewhat agree　　　　Somewhat disagree　　　　Disagree

6－2 Our workplace has an environment of mutual respect and understanding between colleagues.

Agree　　　　Somewhat agree　　　　Somewhat disagree　　　　Disagree

6－3 Our workplace practices adequate work-related information sharing.

Agree　　　　Somewhat agree　　　　Somewhat disagree　　　　Disagree

6－4 I feel alive and motivated to work.

Agree　　　　Somewhat agree　　　　Somewhat disagree　　　　Disagree

6－5 I take pride in my work.

Agree　　　　Somewhat agree　　　　Somewhat disagree　　　　Disagree

1. Please rate your level of satisfaction by choosing one of the options. (2 items)

7－1 Are you satisfied with your work?

Satisfied 　Somewhat satisfied 　Somewhat unsatisfied 　　Unsatisfied

7－2 Are you satisfied with your family life?

Satisfied 　Somewhat satisfied 　Somewhat unsatisfied 　　Unsatisfied

Q2 This section asks you questions about the Walking Intervention.

Please select all the items that describe your motives for participation in the Intervention, and your opinions about the format of the inter-company team challenge.

1. Please select all the motives for your participation in this Intervention.

　I was invited by a manager/superior to participate

　I was invited by a colleague to participate

　I was already thinking about going on a diet

　The inter-company aspect of the challenge was interesting.

　The team challenge aspect was interesting

　The prizes and awards seemed attractive

　Interest in the health event by the CARADA app

　Other (Please describe specifically)

1. Please select all the opinions you may have about the format of the inter-company and team challenge.

<Inter-company competition >

　Company rankings helped maintain my motivation in the race

　Participation as a member of the company helped maintain my motivation in the race

　Inter-company challenge helped build camaraderie

　Increased my company’s interest in measures to improve employees’ health

　Increased my interests in other participating companies

　Could not drop out of the Intervention out of workplace pressure

　Nothing in particular

　Other (Please describe specifically)

<Team competition >

　Team rankings helped maintain my motivation in the race

　Participation as a member of the team helped maintain my motivation in the race

　Team competition helped build camaraderie

　Increased frequency of my social interactions with my team members

　Increased frequency of my social interactions with participants from my company in other teams

　I could not drop out of the Intervention out of team pressure

　Nothing in particular

　Other (Please describe specifically)

1. Please select the option that best describes your team. （4 items）

10－1　Did the frequency of communication with your colleagues increase during the Intervention?

　Yes, increased a lot

　Yes, increased a little

　No, did not increase much

　No, it did not increase at all.

10－2　How many times did you communicate with your team members during the challenge? Please select one from the following options.

　Everyday

　3 to 4 times a week

　1 to 2 times a week

　1 to 2 times a month

　Rarely

10－3　How many people were in your team?

　　　　　　　　　　　　　people

10－4　How was your team formed? Please select from **one** of the following options.

　The team was formed voluntarily by members whom I already knew from work, such as colleagues or those who joined the company in the same year as me.

　The team was formed voluntarily by members whom I socialize with outside of work hours, such as co-members of company clubs or colleagues I eat lunch with.

　The team was formed voluntarily but by members whom I rarely interact with.

　The team members were assigned by a manger/superior, but the team was formed by workers who already knew each other in work-related contexts, such as colleagues or those who joined the company in the same year as me.

　The team members were assigned by a manager/superior, and included members who rarely interacted with one another.

　Other (Please describe specifically)
