# Supplementary material for: Maintaining Physical Activity Level Through Team-Based Walking With a Mobile Health Intervention: Cross-Sectional Observational Study
Source: JMIR Mhealth Uhealth. 2020 Jul 3;8(7):e16159. doi: 10.2196/16159 (PMC7367537; doi:10.2196/16159)
Supplement: Multimedia Appendix 2 [file mhealth_v8i7e16159_app2.docx]

|  | Completers (n=203) | Non-completers (n=213) | P-value^a^ |
| --- | --- | --- | --- |
| age (mean(SD)) | 41.3(8.9) | 38.4(8.5) | .001 |
| % of females | 21.2% | 13.1% | .04 |
| The number of recorded walking steps (mean(SD)) | 8024(3197) | 6643(2796) | <.001 |

^a^The P-values were estimated by t-test (age, number of recorded walking steps) or by chi-square test (% of females).
